# Integration of Traditional, Complementary, and Integrative Medicine in the Institutionalization of Evidence-Informed Decision-Making: The World Health Organization Meeting Report

**DOI:** 10.1089/jicm.2024.0837

**Published:** 2025-01-17

**Authors:** Amie Steel, Daniel F. Gallego-Perez, Nadine Ijaz, Alana Gall, Mukdarut Bangpan, Laura dos Santos Boeira, Mariana Cabral Schveitzer, Anchalee Chutaputti, Laurenz Mahlanza-Langer, Geetha Krishnan G. Pillai, Tipicha Posayanonda, Kim Sungchol, Darshan Shankar, Tanja Kuchenmüller

**Affiliations:** 1 Faculty of Health, Australian Research Consortium in Complementary and Integrative Medicine, University of Technology Sydney, Sydney, Australia; 2 Physical Medicine and Rehabilitation Department, University of North Carolina at Chapel Hill, Chapel Hill, North Carolina, USA; 3 World Health Organization (WHO), Geneva, Switzerland; 4 Department of Law and Legal Studies, Carleton University, Ottawa, Canada; 5 World Federation of Public Health Associations Indigenous Working Group, Geneva, Switzerland; 6 Traditional, Complementary and Integrative Healthcare Coalition, Bruxelles, Belgium; 7 Social Research Institute, University College London, London, United Kingdom; 8 Veredas Institute, São Paulo, Brazil; 9 Brazilian Academic Consortium of Integrative Health (CABSIN), Universidade Federal de São Paulo, São Paulo, Brazil; 10 Department of Thai Traditional and Alternative Medicine, Ministry of Public Health, Bangkok, Thailand; 11 Pan-African Collective for Evidence (PACE), Johannesburg, South Africa; 12 Knowledge and Innovation Management Department, National Health Commission Office, Bangkok, Thailand; 13 Trans-Disciplinary University, Bangalore, India

## Background and Context

The use of evidence in policy and decision-making has exponentially grown, and it is now considered standard practice within health systems.^1,2^ However, the gap between research and practice persists. Seeking to translate health research findings into policy and practice, the World Health Organization (WHO) has advanced initiatives that promote the institutionalization of Evidence-informed decision/policy-making (EIDM).^3,4^ The WHO Secretariat of the Evidence-Informed Policy Network (EVIPNet) has developed an EIDM institutionalization checklist that provides a structuring framework.^5^ The checklist, currently pilot-tested to assess its validity and feasibility,^6^ highlights six domains (governance; standards and routinized processes; leadership and commitment; resources and capacity-building/strengthening; partnership, collective action, and support; and culture) and five processes of EIDM institutionalization.^5^

Both EIDM and the structuring of health systems based on Primary Health Care (PHC) have been recognized as essential for advancing toward universal health coverage and health-related Sustainable Development Goals.^5,7,8^ PHC is a whole-of-society approach that was initially characterized as context-specific, based on research and experience, health needs-focused, intersectoral, and delivered by health teams that include traditional medicine practitioners.^9^ The Alma Ata conference recommended studying the contribution of “traditional systems of medicine.”^9^ The Astana Declaration, in turn, recognized the importance of traditional knowledge, in conjunction with scientific knowledge, to “strengthen PHC, improve health outcomes and ensure access for all people to the right care.”^8^

Despite this recognition and the documented use of traditional, complementary, and integrative medicine (TCIM) by 88% of WHO Member States,^10^ countries have identified significant gaps in realizing TCIM’s potential contributions to improving health outcomes and well-being.^10^ They have requested that the WHO prioritize evidence and data to inform policies, standards, and regulatory frameworks for the safe, cost-effective, and equitable use of TCIM.^10,11^

The Gujarat Declaration of the first WHO Global Summit on Traditional Medicine (August 17 and 18, 2023, Gandhinagar, India) articulated an action agenda including a focus on research and evidence. It proposed “making appropriate use of existing and new research, evidence syntheses and knowledge translation principles and WHO initiatives.”^12^ It also recommended capacity strengthening “to produce, translate and use TCIM research and Indigenous knowledges” and supporting “the evidence-based integration of TCIM in national health policies and systems based on the highest quality research.”^12^

The recently established WHO Global Traditional Medicine Center (GTMC) is tasked with augmenting WHO’s capacities for mobilizing knowledge for policies and standards for TCIM practices and products. The GTMC will, in collaboration with WHO technical departments, implement the Gujarat Declaration proposals, complementing the core WHO functions of governance, norms, and country support carried out by its technical unit on TCIM and the six regional offices. Furthermore, the WHO is developing a new TCIM global strategy (2025–2034)^13^ that will incorporate EIDM processes.

To support these goals, the WHO Evidence to Policy and Impact Unit, in collaboration with the Research and Evidence Unit of the WHO’s GTMC, hosted a side event at the 2024 Prince Mahidol Award Conference (PMAC) in Bangkok, Thailand. The side event explored the current state of EIDM’s institutionalization globally and the implications of its intersections with TCIM in fostering inclusivity, health equity, epistemic justice, and decolonial global health governance (see Table 1). Side event participants, who represented multiple world regions, explored potential mechanisms (infrastructure, conditions, and frameworks) for enhancing the use of evidence in global policy development toward realizing TCIM’s contribution to health and well-being. This article presents a synthetic account of the event’s discussed issues.

## EIDM and TCIM: What Evidence? What Knowledges?

The presenters at PMAC’s side event discussed a series of distinct considerations that arise at the interface of EIDM’s institutionalization and TCIM. Foremost among these are questions pertaining to what counts as evidence—and ultimately, what counts as legitimate knowledge—in the EIDM process.

From the WHO’s own definition,^11^ traditional medicine is the sum total of the knowledge, skill, and practices based on the theories, beliefs, and experiences [I]ndigenous to different cultures, whether explicable or not, used in the maintenance of health as well as in the prevention, diagnosis, improvement or treatment of physical and mental illness.

As this definition indicates, a key characteristic of TCIM therapeutic approaches, which are often not “fully integrated into the dominant [biomedical] health-care system,”^11^ is that they are in many cases underpinned by “medical rationalities,” that is, systems of therapeutic knowledge and practice, that differ distinctly from dominant biomedical science.^14^ Such therapeutic systems include Indigenous healing systems characterized by orally transmitted knowledges; codified ethnomedical systems like Chinese and Ayurvedic medicine; as well as codified whole systems like naturopathy, anthroposophy and osteopathy.^15^ Like biomedicine, each of these systems has its own underlying cosmology, anatomical and physiological models, medical doctrine as to how health and ill-health arise, and diagnostic and therapeutic systems.^14^ They are, in other words, distinct systems of therapeutic knowledge and practice in their own right, despite not having the same degree of social, economic and political capital as does biomedical science today.

As historians and anthropologists have carefully documented, biomedicine is a relatively young therapeutic system that developed in 17th-century Europe.^15^ Along with its many notable therapeutic contributions, biomedicine was used as a tool of empire, as part of the European colonial encounter, through which many traditional and Indigenous medical systems and practices were subjugated and, in some cases, decimated. Biomedicine’s globalized political dominance today, then, is at least partly built upon this history. Further, as Cloatre observes:^16^

With colonial expansion, and as biomedicine became a powerful tool of domination and population control, the separation of “knowledge” and “belief,” or “rational” and “irrational,” was central to the settlement of socio-political power.

In other words, the persistence of biomedicine’s global dominance is predicated at least partly on its discursive self-presentation as uniquely rational and universal despite the notable diversity of therapeutic rationalities that persist across cultures and regions.

Further, the research methods that underpin today’s ‘evidence-based medicine’ movement are substantially aligned with the biomedical paradigm and can fall short in rigorously studying many TCIM approaches, whose underlying paradigms differ considerably. This problem of ‘paradigmatic (dis)alignment’ is currently the focus of a WHO-commissioned evidence review of TCIM research approaches.^17^

To align with the WHO’s repeated calls for traditional and Indigenous knowledges to be recognized in the TCIM integration process,^11^ advocates of EIDM’s institutionalization in this context must contend with the realities of therapeutic knowledge diversity (or, “epistemic pluralism”). The concept of ‘epistemic justice’ is often used, including by the United Nations Educational, Scientific and Cultural Organization (UNESCO), to characterize a decolonial construct that is ultimately a “condition for realizing social and environmental justice.”^18^ Epistemic justice, in the TCIM context, may be understood as a “two-fold call for: a) the equitable engagement, within health systems, of biomedical as well as TCIM paradigms — both at the level of knowledge and practice; and b) the respectful and socially-just recognition of the perspectives and contributions of community members, knowledge holders, and health care professionals alike.”^15^

This pursuit of epistemic justice is closely reflected in the work of Nugruho and colleagues,^19^ who have outlined three major categories of knowledge important to decolonial EIDM. These are: (1) scientific knowledge (from multiple research approaches), (2) professional knowledge (across various communities of expertise), and (3) local knowledge (from community stakeholders). Ultimately, such an approach is aligned with the principles of evidence-based medicine (which emphasize scientific evidence, clinical experience and peoples’ preferences and values), but it requires a broader conceptualization of what each of these tenets may mean in practice. In the context of TCIM, how may TCIM knowledges be appropriately and respectfully translated for use in decision-making processes?

With respect to research, effective knowledge translation means ensuring that scholarly findings are not only published but are also communicated in a way that facilitates their integration into clinical practice, education, and policy. TCIM knowledge translation also requires that TCIM research is conducted in a way that effectively honors and represents TCIM knowledges at all stages. Following Nugruho et al.’s decolonial guidance for EIDM,^19^ it would also require active engagement with diverse stakeholders across sectors to adapt TCIM knowledge into different health care contexts while respecting cultural nuances, redressing historical inequities, and addressing potential barriers to implementation.

One useful knowledge translation framework developed in the TCIM field is the Contemporary Implementation of Traditional Knowledge and Evidence (CITE) Framework.^20,21^ As shown in Table 2, the framework includes guiding principles and criteria to support rigorous and respectful translation of codified forms of TCIM knowledge into contemporary research, education, policy and practice. However, how to apply—or rather, adapt—the Framework with reference to orally-transmitted TCIM approaches, such as Indigenous Peoples’ traditional medicine, illustrates the complexity of EIDM’s institutionalization in the TCIM field.

## TCIM Knowledge Translation for Indigenous Traditional Medicines

Translating Indigenous Peoples’ traditional medicine knowledges within an EIDM context—where codified knowledges are taken as normative—poses distinct challenges. One reason for this is that around the world, Indigenous knowledges are primarily communicated and sustained through oral rather than textual transmission. The CITE Framework authors noted it was “primarily designed for use with knowledge from traditional medicine systems with an established history of written traditional sources.”^20^ Thus, to be fruitfully applied with reference to orally transmitted knowledges, it would require adaptation.

Key considerations for adapting the CITE Framework, consistent with principles affirmed in the UN Declaration on the Rights of Indigenous Peoples,^22^ include attention to issues of free, prior, and informed consent and the equitable sharing of benefits arising from usage of Indigenous traditional medicine approaches, with the communities from which related knowledges originates.^22,23^ It also warrants note that within Indigenous communities, traditional medicines often carry not only ‘therapeutic’ value but also cultural, spiritual and ecological meaning as part of a way of life. Further, any process of knowledge evaluation would optimally be led by Indigenous peoples who have the cultural authority to authenticate particular knowledges, practices, and safety-related claims. The specifics of how such a process might be enacted may also vary across Indigenous communities, each of which has its own authority structures and laws determining who are knowledge holders and community representatives.

Existing EIDM tools and frameworks acknowledge evidence may take ‘non-scientific’ forms, such as ‘tacit’ or informal knowledges, including “opinions, values and habits.”^24^ However, epistemic justice principles call for expanding our understanding of what constitutes legitimate forms of evidence and knowledges in considering how to incorporate traditional knowledge (and TCIM) in EIDM. Further conceptual and applied developments are thus warranted. Examples can be found in several countries, whereby decision-makers have taken steps to incorporate both scientific and orally transmitted knowledges into EIDM processes.

## Case Examples of EIDM Institutionalization for TCIM

PMAC side event presenters reported on international case examples from Thailand and Brazil that demonstrate opportunities and successes in implementing EIDM institutionalization for TCIM.

### Thailand

Thailand has implemented a citizen engagement process to support the integration of TCIM-based EIDM into institutions. This process was used to draft the National Health Act, which was enacted in 2007 and led to the development of the institutional structure and a National Health System Charter to support drafting of health-related policies and strategies, including TCIM, that focus on good health system governance. Moreover, since Thailand’s health system reform in the 1990s, several laws related to Thai traditional medicine have been enacted.^1^

The Charter explicitly commits to ‘promoting, supporting, utilizing and developing health wisdom, Thai traditional medicine, [I]ndigenous medicine and other alternative medicines’ (Section 46–48, National Health Act 2007). Its implementation enabled the government to better support participatory processes by funding, monitoring and evaluating public servant training and formal social participation structures that integrate traditional wisdom, including health policy and planning processes. The Charter also led to policy recommendations which could drive funding supports on TCIM medical services and related issues. Consequently, the percentage of outpatients receiving Thai traditional and alternative medical services and the number of research studies on Thai traditional and alternative medicines have grown.

Thailand has also employed institutionalized EIDM to the process of developing a National List of Essential Herbal Medicines (NLEHM) that includes three different categories: (1) Thai Traditional medicines, (2) Thai Indigenous medicines, and (3) Herbal medicines. This initiative recognizes traditional medicines based on written classical texts as well as oral knowledge from Indigenous healers adopted, manufactured, and dispensed by community hospitals. It also provides some acknowledgment and guidance of traditional medicines that have been modified from traditional use (e.g., by modifying manufacturing process or dosage form) and the process for recognizing scientifically established herbal medicines. More than 100 traditional and herbal medicines have been selected through this process into the NLEHM and covered by the three health security systems of Thailand. Further, community pharmacists have been provided training to ensure they can safely dispense selected items in the NLEHM for common illnesses.

### Brazil

The Brazilian Coalition of Evidence (https://coalizaopelasevidencias.org.br/) is a network of academic, government and civil society representatives working within or committed to evidence translation and dissemination across Brazil with a particular focus on social policies. The Coalition applies tools developed by EVIPNet such as the Situation Analysis Manual and the WHO Checklist for EIDM. Brazil has seen numerous institutionalized evidence units open since 2018, and the Coalition has undertaken an exploratory study of EIDM institutionalization.

In parallel, a collaboration of TCIM researchers across the Americas region (i.e., TCIM Americas Network, the Brazilian Academic Consortium for Integrative Health, and the Pan American Health Organization/WHO’s Latin American and Caribbean Center on Health Sciences Information) has adapted an evidence-mapping method to synthesize and translate TCIM research for EIDM among health professionals, decision-makers, and researchers. The Evidence Map methodology involves a six-step process of (1) Search, (2) Selection, (3) Categorization, (4) Infometrics, (5) Evidence Map, and (6) Gaps. The Maps resulting from this process present a matrix that provides an overview of the evidence pertaining to particular TCIM interventions and specific health outcomes.^25^

To date, the Evidence Map team has produced 26 TCIM Evidence Maps derived from more than 2000 systematic reviews, which are increasingly influencing TCIM-related decision-making in Brazil. In 2020, for example, Brazil’s national health council issued guidance advising the country’s health systems actors to review TCIM-related evidence produced by the aforementioned Evidence Map collaboration in developing interventions for COVID-19.^26^ Further, in 2023, the city of Sao Paulo released clinical guidance for chronic pain informed by a related TCIM Evidence Map.^27^ Currently, the Pan American Health Organization is reviewing the Evidence Maps to include TCIM-related interventions in its technical co-operation activities. Some Evidence Maps are also now being translated into public education materials.

### Discussion and workshop recommendations

Following a series of expert presentations, side event workshop participants discussed the content of the presentations and proposed some areas that required further consideration. As shown in Table 3, these discussions centralized the following issues: terminology and concepts; technology and infrastructure; capacity and governance; TCIM-appropriate research methods; and implementation and dissemination. Insights across these broad topics offer initial guidance toward addressing the aims of the event (see Table 1) and will support the activities of the WHO’s GTMC more broadly.

A noteworthy aspect of these recommendations is the implicit acknowledgment that research alone is not sufficient for the effective integration of TCIM. Rather, a multipronged process of translating knowledge into practice is needed. Such a process necessarily involves the synthesis, dissemination, exchange, and ethically sound application of knowledge to improve health services, provide more effective health care, and strengthen health systems.

EVIPNet is an example of a WHO initiative that has been developing EIDM frameworks and tools for effective knowledge translation in closing the evidence-to-policy gap. Systematic inclusion of TCIM and traditional knowledge in EIDM processes, however, would require an expansion of the conceptual and operational hallmarks used thus far in the said frameworks and tools. As highlighted in the Gujarat Declaration, EVIPNet is uniquely positioned for catalyzing the required developments to make it possible and, with appropriate engagement with TCIM experts and community leaders, may provide the infrastructure and support needed to achieve EIDM institutionalization for TCIM globally.

## Conclusion

This meeting highlights the pressing need to foster an intersection between EIDM institutionalization and TCIM. It is critical that efforts in this regard align with decolonial principles of epistemic justice and knowledge diversity. Moving forward, those seeking to advance EIDM institutionalization initiatives related to TCIM would wisely consider conceptual frameworks and models that are tailored to the unique features of TCIM worldviews, knowledges and practices. The meeting outcomes offer conceptual guidance, case examples, and initial reflections for what is undoubtedly a long and complex road ahead.

## Figures and Tables

**Table 1:** Aims of the WHO-Hosted Side Event of the Prince Maldihol Award Conference in Bangkok, Thailand, 2024

Explore how the application of EIDM in the TCIM context could foster inclusivity, health equity, epistemic justice, and decolonial global health governance. Assess the advances and challenges of integrating TCIM in EIDM institutionalization globally and the needed conditions to strengthen it. Outline potential next steps for implementing the evidence-related proposals of the Gujarat Declaration, particularly regarding the “evidence-based integration of TCIM in national health policies and systems based on highest quality research” and advancing complementarities with existing WHO-led EIDM initiatives.

EIDM, evidence-informed decision-making; TICM, traditional, complementary and integrative medicine; WHO, World Health Organization.

**Table 2:** The Contemporary Implementation of Traditional Knowledge and Evidence Framework (Steel et al.^20^)

Section 1: Guiding principles for the contemporary use of traditional knowledge Accountability during translation of knowledge Importance of foundational assessment of traditional knowledge Evolution of practice in living traditions Pragmatic translation to balance traditional perspective and contemporary context Peer-to-peer knowledge sharing and empirical observation
Section 2: Criteria for critically appraising traditional knowledge sources Authenticity of the traditional knowledge source Consistency of evidence across sources Safety of the traditional knowledge in the contemporary context
Section 3: Criteria to guide the application of traditional knowledge in contemporary settings Alignment with core characteristics of the tradition Ethical approaches: intellectual property and sociological considerations Tradition-informed communication and framing Person-centered translation Accuracy of interpretation Transferability of traditional knowledge to contemporary context Accessibility and integrity of traditional resources Comparative benefit between available traditional and non-traditional approaches

**Table 3:** Recommendations and Insights Arising from the Workshop Discussion

	
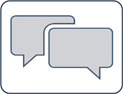	**TERMINOLOGY AND CONCEPTS**
	Develop terminology and conceptual frameworks that makes TCIM comprehensible without requiring it to retrofit to the biomedical lens Address the diversity of classifications and terminology used with reference to: - different types of TCIM therapeutic approaches - different TCIM therapeutics used for the same biomedical diagnosis - different countries for the same TCIM system or practice
	
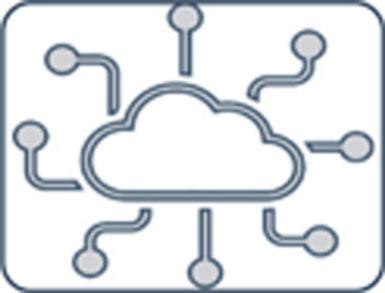	**TECHNOLOGY AND INFRASTRUCTURE**
	Use advanced technologies such as artificial intelligence Build a global repository of TCIM knowledges, evidence, and policy Design (or tailor existing) data systems to collect TCIM practitioner knowledges (e.g., case report portals) ICD Module 1 & 2 and integration with RHIMS Develop platforms to support researcher, practitioner, and policymaker collaboration
	
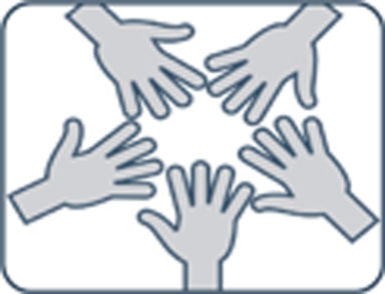	**CAPACITY & GOVERNANCE**
	Foster culturally-reponsive leadership, champions of knowledge translation, and research skills development for TCIM Develop TCIM-appropriate and protective governance mechanisms and regulatory pathways Include TCIM policy-makers and practitioners in knoweldge translation training opportunities Funding for TCIM research (generation and translation) and practice within health systems Develop national participatory mechanisms that facilitate co-production, shared decision- making, autonomy and power, inclusive of communication between TCIM groups
	
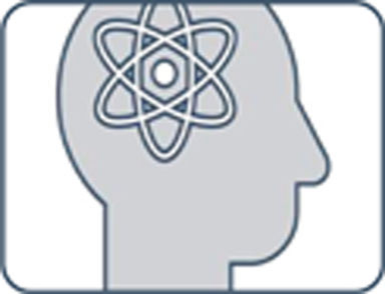	**TCIM-APPROPRIATE RESEARCH METHODS**
	Prioritise and innovate research approaches that align with TCIM knowledge paradigms (e.g., evaluates complex interventions and 'whole systems', incorporates TCIM diagnoses, accounts for herbal complexity) Foster Indigenous research methodologies and their translation, including decolonial scholarly frameworks Reevaluate evidentiary hierarchies and make use of research rigorously conducted across diverse disciplines, methodologies and methods Support practice-based evidence and complexity science
	
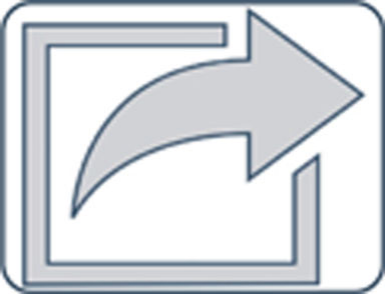	**IMPLEMENTATION AND DISSEMINATION**
	Support mechanisms/methods to integrate different knowledge types Build TCIM knowledge across biomedical practice and research communities Foster Indigenous-led health care partnerships that incorporate TCIM knowledges and EIDM Provide TCIM-inclusive community health education platforms, that involve local TCIM systems, practitioners and networks Develop mandates and approaches for TCIM inclusion in EIDM
	

EIDM, evidence-informed decision-making; TICM, traditional, complementary and integrative medicine; ICD, International Classification of Diseases

## Footnotes

1 Protection and Promotion of Thai Traditional Medicine Knowledge Act B.E. 2542 (1999), National Health Security Act B.E. 2545 (2002), Laws on the establishment and departmental organizational structure of the Department for Development of Thai Traditional and Alternative Medicine B.E. 2545 (2002), Thai Traditional Medical Professions Act B.E. 2556 (2013).
